# Physical Injury and Psychological Impact: Understanding the High Risk of Depression on Older Adults with Recurrent Falls

**DOI:** 10.20900/agmr20240008

**Published:** 2024-12-18

**Authors:** Asmaa Namoos, Nicholas Thomson, Carol Olson, Michel Aboutanos

**Affiliations:** Department of Surgery, Medical Center West Hospital, Injury and Violence Prevention Program, School of Medicine, Virginia Commonwealth University, Richmond, VA 23219, USA

**Keywords:** falls, recurrent falls, depression, anxiety, older adults, retrospective study, risk factors, TriNetX

## Abstract

**Background:**

Falls among older adults significantly increase the risk of physical injuries, loss of independence, and social isolation, contributing to psychological conditions such as depression.

**Objective:**

This study explores the association between falls and the risk of developing depression among older adults, comparing psychological outcomes between those with initial and recurrent falls. It also examines demographic factors such as age, sex, and race that may influence depression risk.

**Methods:**

We retrospectively analyzed electronic health records from the TriNetX network at Virginia Commonwealth University Health System (VCUHS) during 2023. Older adults aged 65 to 89 were classified into two cohorts: initial falls (*n* = 2710) and recurrent falls (*n* = 1050). Statistical analyses, including risk ratios, survival analysis, and proportional hazards models, were used to evaluate associations between falls and depression risk.

**Results:**

Recurrent fallers exhibited a higher prevalence of depression (25.7%) compared to initial fallers (16.6%), with a significant association (*p* < 0.000). Recurrent fallers were 48.8% more likely to develop depression (HR = 1.488). Among younger adults aged 65 to 69, females had a higher prevalence of depression than males (30.5% vs. 20.1%). Anxiety disorders tripled the risk of depression following falls (HR = 3.036).

**Conclusions:**

Recurrent falls are significantly associated with an increased likelihood of depression among older adults, highlighting the need for comprehensive interventions. Preventing falls not only reduces the risk of physical injuries but also alleviates associated mental health challenges, including depression and other comorbidities such as postoperative cognitive decline and dementia. Tailored prevention strategies, such as balance training, cognitive therapy, and home safety modifications, can foster better recovery and enhance the quality of life for this vulnerable population.

## INTRODUCTION

Depression in older adults across the United States is a pressing yet often hidden issue, affecting nearly one in ten individuals aged 65 and up [1]. Studies indicate that around 15% of older women report symptoms of depression, compared to about 7%–10% of older men [2]. Women are more likely to experience emotional distress due to life events such as the loss of a spouse or caregiver responsibilities, which increases their vulnerability to depression [3]. Depression in older men, when untreated, is associated with a higher risk of suicide compared to women [4]. Despite its prevalence, depression frequently goes undetected in this age group, partly due to overlapping symptoms with other health conditions and the social stigma that discourages open discussion [5]. Physical limitations, chronic health conditions, and sensory impairments compound the risk, contributing to a sense of isolation and diminishing independence [6]. Additionally, life changes such as retirement or the loss of loved ones add to emotional challenges [7].

The human body, while resilient, becomes increasingly vulnerable with age, and nowhere is this more evident than in the growing risk of falls among older adults [8]. According to the CDC’s Behavioral Risk Factor Surveillance System, approximately 14 million older adults aged 65 and older in the United States reported experiencing a fall in 2020. This represents about 28% of the population in this age group, meaning nearly “1 in 4” older adults had a fall during the previous year ([9], p. 2020–2021).

The relationship between physical limitations due to falls and depression is particularly concerning as it creates a vicious cycle: depression can lead to reduced physical activity, worsening mobility, and social withdrawal, which in turn increases the risk of falls [10,11]. Conversely, experiencing a fall can lead to fear of falling, reduced activity, and subsequent depression [12]. Recurrent falls, defined as two or more falls within a year, pose an even greater risk, not only exacerbating the likelihood of severe injuries but also increasing the potential for developing or deepening depression [13].

Despite extensive research on fall prevention and management, less attention has been given to the psychological outcomes following falls, especially the differential impacts of initial versus recurrent falls on mental health. This study aims to fill that gap by exploring the association between fall frequency (initial vs. recurrent) and the risk of developing depression in older adults, utilizing a large-scale retrospective dataset from the TriNetX network at Virginia Commonwealth University Health System (VCUHS). This approach not only highlights the immediate effects of falls on physical health but also underscores the longer-term psychological challenges, paving the way for integrated interventions that can improve both physical and mental health outcomes for older adults.

## METHODS

### Study Design

This is retrospective study focusing on older adults aged 65 to 89 who experienced either an initial or recurrent fall in 2023. The objective is to explore the relationship between physical injuries from falls and the risk of developing depression, comparing psychological outcomes between individuals with initial versus multiple fall incidents. The study utilizes electronic health records (EHRs) from the TriNetX network at Virginia Commonwealth University Health System (VCUHS) and leverages detailed demographic and clinical data from this specific year.

### Data Source

TriNetX is an extensive health research network that links healthcare organizations with life sciences companies. It offers real-world data to support clinical research and the discovery of new treatments. The platform grants researchers access to anonymized and aggregated patient data from electronic health records (EHRs), facilitating observational studies, optimizing clinical trials, and generating evidence-based insights to drive advancements in healthcare [14].

### Data Collection

Data were extracted from the electronic health records (EHRs) available through the TriNetX network at Virginia Commonwealth University Health System (VCUHS). The dataset includes detailed demographic information, such as age, sex, race, and ethnicity, along with ICD-10 diagnosis codes [15]. Additionally, it records information on falls, recurrence of falls, types of depression. The dataset also captures the timing and severity of falls, hospital visits related to these incidents, and clinical history relevant to fall risk factors.

### Patient Selection

Patients in this study were categorized into two cohorts: those experiencing an initial fall and those with recurrent falls within one year. The ICD-10 codes used in this study are based on the World Health Organization’s (WHO) International Classification of Diseases and capture various types of falls and related conditions, as outlined in Table 1 below:

### Handling of Multiple Records and Missing Data

In this study, each patient was analyzed individually. If a patient had multiple fall incidents during the study period, only the first recorded fall was included in the primary analysis. Any additional falls that occurred more than one year after the initial incident were documented as recurrent falls but were not treated as distinct events for analysis purposes.

The TriNetX platform ensures high standards of data quality and completeness. Consequently, the dataset utilized in this study had no missing values for key variables, including diagnosis codes and demographic information, allowing for comprehensive analysis without the need for data imputation [14].

#### For missing data

The TriNetX platform aggregates real-world data from electronic health records (EHRs) across healthcare organizations, ensuring that all necessary patient data are complete and de-identified. TriNetX’s federated data network maintains rigorous data governance protocols, minimizing the occurrence of missing data. Therefore, for this analysis, there were no missing data for key variables such as diagnosis codes, demographic characteristics, or clinical outcomes.

### Incidence and Prevalence Calculation

Incidence proportion in TriNetX is the rate of new or first-time cases within a cohort during a given time window. Patients are included in the denominator if their records overlap with the time window, and they have no prior diagnosis of the event of interest within a set lookback period. The incidence rate accounts for the time at risk, calculated by multiplying the incidence proportion denominator by the number of days in the time window. Both the numerator and denominator align with those used for the incidence proportion. Prevalence reflects all cases within or before the time window. The prevalence denominator includes patients whose records overlap the time window, and the numerator includes those with a relevant diagnosis either before or during the time window. These calculations follow TriNetX’s established framework for analyzing real-world EHR-based data [16].

### Statistical Analysis

All analyses were conducted using the TriNetX platform, which provided robust tools for data extraction, analysis, and visualization. This ensured consistency and accuracy in evaluating the relationship between falls and subsequent health outcomes among older adults.

Descriptive statistics summarized the cohorts’ baseline characteristics, providing an overview of age, sex, and other demographic factors. Baseline comparison statistics, including chi-square tests for categorical variables and t-tests for continuous variables, examined initial differences between groups.

To explore associations between falls and subsequent health outcomes, we calculated risk ratios, risk differences, and odds ratios, each accompanied by 95% confidence intervals to quantify the strength and precision of the observed associations. For survival analysis, the Kaplan-Meier method was used to estimate time-to-event data, such as the time from an initial fall to the onset of depression. Survival distributions between groups were compared using log-rank tests, and censoring was applied at the last recorded observation for each patient to account for incomplete follow-up.

**Figure 1.**
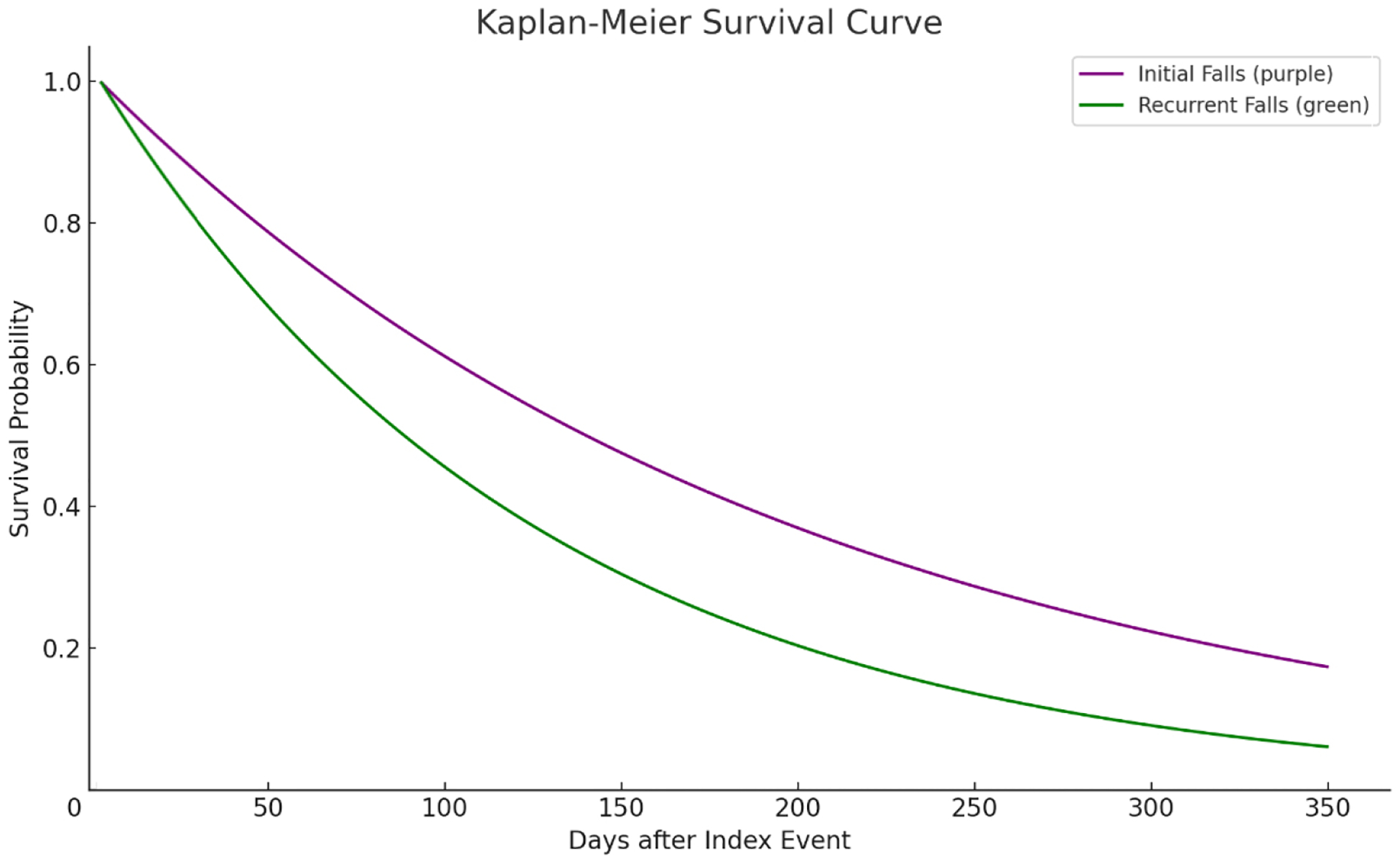
Kaplan Meier Curves comparing depression risk after initial and multiple falls [14].

Hazard ratios (HRs) were derived using Cox Proportional Hazards Models to evaluate the relative likelihood of developing depression associated with recurrent falls compared to initial falls. A hazard ratio greater than 1 indicates an increased likelihood of the outcome, while a ratio less than 1 indicates a decreased likelihood. Kaplan-Meier survival curves were used to visually depict these temporal relationships, providing insights into differences between cohorts over time. This multifaceted statistical approach ensured a robust analysis of the interplay between falls and depression in older adults. The visual figure (Figure 1) was completed through the TriNetX platform, and the copyright for the figures belongs to them.

### Software and Tools

All analyses were conducted using the TriNetX platform’s analytical tools. These tools enabled processing of large datasets and provided robust statistical evaluations and visualizations to support epidemiological analysis.

## RESULTS

### Demographic Characteristics

This study focuses on examining demographic and psychological characteristics following a fall in older adults, aged 65 to 89, who experienced falls in 2023. The participants were divided into two cohorts: Cohort 1, comprising 2710 individuals who experienced an initial fall, and Cohort 2, consisting of 1050 individuals with recurrent falls within the same year. The average age in Cohort 1 is 76.1 years (mean ± SD: 76.1 ± 6.87; range: 65 to 89 years). Similarly, Cohort 2 has an average age of 76 years (mean ± SD: 76 ± 6.83). Statistical tests confirm that the difference in age between the two groups is not significant, with a *p*-value of 0.7023. This trend holds when considering the age at the index fall, where Cohort 1 has a mean age of 74.8 years and Cohort 2 has a mean age of 74.6 years, also yielding no significant difference (*p* = 0.5622). The sex distribution within the study population, Cohort 1 included 1580 females (58.3%) and 1140 males (41.7%). In contrast, Cohort 2 had 590 females (56.2%) and 470 males (44.8%). Statistical analysis shows that the difference in sex distribution between the two cohorts is not significant, with a *p*-value of 0.2395, indicating a similar Sex makeup across both groups.

Racially, we found that 64% of Cohort 1 and 68% of Cohort 2 are White, with the difference being statistically significant (*p* = 0.0488). This indicates a slightly higher prevalence of Whites in the cohort with recurrent falls. In contrast, the proportions of Black or African American participants are relatively similar across the cohorts, at 31% for Cohort 1 and 28% for Cohort 2, with the difference not proving statistically significant (*p* = 0.0704). Additionally, Native Hawaiian or Other Pacific Islanders show a noticeable difference, completely absent in Cohort 1 but representing 1% of Cohort 2, with this difference being highly significant (*p* < 0.0001) (Table 2).

### Incidence and Prevalence

Table 3 details the incidence and prevalence rates for older adults who experienced an initial fall and subsequently exhibited signs of depression, broken down by age, sex, and race. Overall, the incidence proportion is 8.955%, the prevalence is 32.472%, and the incidence rate is 0.000353 cases per person-day. Notably, the age group 65–69 years shows the highest incidence proportion at 0.1163 and prevalence at 0.3559, indicating a significant risk of depression following an initial fall in this demographic. The incidence rate decreases with advancing age, with the 75–79 age group recording an incidence proportion of 0.0682 and prevalence of 0.3167, suggesting that while older adults may experience fewer new cases, the enduring impact of depression remains substantial across all age brackets. Females have a higher prevalence rate of 0.3734 compared to males, who show a prevalence of 0.2632, although males exhibit a slightly higher incidence rate of 0.000399 per person-day versus 0.000351 for females. Racial disparities are also evident; the ‘Other Race’ category shows a strikingly high incidence proportion of 0.25 and prevalence of 0.4, which significantly outpaces other groups. White participants report an incidence proportion of 0.1016 and a prevalence of 0.3391, whereas Black or African American individuals have lower rates, with an incidence proportion of 0.0656 and a prevalence of 0.3133.

Table 4 presents a comprehensive analysis of incidence and prevalence rates for older adults who experienced recurrent falls and subsequent depression, Overall, the incidence proportion is 16.418%, the prevalence is 45.192%, and the incidence rate is 0.000687 cases per person-day. Notably, the age group 65–69 shows the highest incidence proportion at 0.2143 and a substantial prevalence of 0.5455, suggesting that individuals in this age bracket are particularly vulnerable to depression following recurrent falls. As age increases, both the incidence proportion and prevalence generally decrease, with the 85 and older group showing the lowest rates. Females exhibit a higher incidence proportion and prevalence (0.1818 and 0.5345, respectively) than males (0.1429 and 0.3696). Asians show a markedly high incidence proportion and prevalence of 1.0, indicating that every Asian older adult in the cohort experienced depression, which could suggest either a small sample size or high vulnerability. In contrast, American Indians/Alaska Natives and Native Hawaiians/Pacific Islanders show no recorded cases, potentially due to underreporting or genuinely lower rates. White participants and Black or African Americans show more moderate figures, with prevalence rates of 0.4857 and 0.4483, respectively, reflecting broader trends in the general population. The ‘Other Race’ category and ‘Unknown Race’ group show notably higher rates than most other races, which could highlight specific vulnerabilities or data anomalies.

### Risk Association

We evaluate the risk of depression in depressive patients following initial and recurrent falls by utilizing the Measures of Association tool in TriNetX. Among the patients who experienced an initial fall, 450 individuals (16.6%) exhibited symptoms of depression. In contrast, 270 individuals (25.7%) in the recurrent fall cohort displayed depressive symptoms. The risk difference between the two cohorts is −9.109%, with a 95% confidence interval from −12.101% to −6.117%, and a highly statistically significant *p*-value (<0.0001). This suggests that the observed difference in depression risk between the two groups is robust and unlikely to be due to chance, as supported by a Z-score of −6.369.

Additionally, the odds ratio for the risk of depressive episodes in Cohort 1 versus Cohort 2 is 0.646, with a 95% confidence interval of 0.565 to 0.738. This significant odds ratio highlights a strong association between recurrent falls and increased depression risk.

Our regression analysis in Table 5, employing the Cox Proportional Hazards Model, examined factors that contribute to the development of depression following a fall in elderly patients, differentiating between those with initial falls (Cohort 1) and those with recurrent falls (Cohort 2). The findings show that individuals in Cohort 2, or those experiencing recurrent falls, have a higher risk of developing depression, evidenced by a hazard ratio (HR) of 1.488. This indicates that they are 48.8% more likely to develop depression compared to those with initial fall, and this association is statistically significant with a *p*-value of less than 0.0001.

Regarding sex differences, the analysis reveals that males have a lower risk of depression after a fall, with a hazard ratio of 0.866. This suggests that males are approximately 13.4% less likely to develop depression than females, although this result is not statistically significant, as indicated by a *p*-value of 0.0687.

Age at the time of the fall also plays a role, with a hazard ratio of 0.987 per year increase. This implies that each additional year slightly decreases the risk of developing depression by about 1.3%, a statistically significant finding (*p*-value = 0.0172).

The presence of an anxiety disorder significantly increases the risk of depression after a fall regardless initial or recurrent, with a hazard ratio of 3.036. This means that patients with anxiety are over three times more likely to develop depression following falls, a relationship underscored by a highly significant *p*-value of less than 0.0001.

The Kaplan-Meier analysis (Figure 1) evaluates the likelihood of developing depression over time among two groups: individuals with initial fall and those with recurrent falls. In the initial fall cohort of 2710 patients, 490 experienced depressive episodes, with a 74.81% probability of remaining depression-free by the end of the study period. In contrast, the recurrent fall cohort, consisting of 1280 patients with 270 depressive episodes, demonstrated a lower probability of 65.69% for remaining free from depression. The survival curve illustrates that those with recurrent falls face a higher risk of depression over time. Supporting this, the hazard ratio is 1.30 (95% CI: 1.08–1.57, *p* = 0.004), indicating a 30% greater risk of depression for individuals with recurrent falls compared to those with initial fall.

## DISCUSSION

In this retrospective study focusing on older adults aged 65 to 89 who experienced either an initial or a recurrent fall in 2023, we have uncovered the significant psychological impact that recurrent falls can have on this population. Our analysis reveals that age, sex, and race significantly influence both the likelihood of experiencing falls and the risk of subsequent depression. While it is well-established that advanced age and female sex are risk factors for initial falls—primarily due to decreased balance, muscle strength, and bone density—these factors do not strongly predict the likelihood of recurrence [17–19]. This suggests that recurrent falls may be more influenced by external factors or specific health conditions beyond aging alone. Interestingly, although females generally exhibit a higher risk of falling due to lower muscle mass and a greater prevalence of osteoporosis, they do not show a marked difference in recurrence rates. The observed sex-based differences in fall risk may also be attributed to biological factors, such as hormonal changes that affect bone density and muscle strength, particularly post-menopause [20]. Social and cultural influences, including caregiving responsibilities and differing levels of physical activity, may further contribute to these disparities. These findings align with literature highlighting the multifaceted nature of sex-based differences in fall risk, warranting further exploration to inform tailored interventions [21].

When considering the psychological aftermath of an initial fall, we find it closely tied to demographic influences, particularly age and sex. Younger elderly adults, especially those aged 65–69, demonstrate the highest incidence and prevalence of depression after a fall. This aligns with existing literature that suggests this age group is particularly vulnerable to depressive symptoms due to the psychological strain of adjusting to early health declines [5,22]. Additionally, sex differences emerge, as females tend to display a higher prevalence of depression following health setbacks. This disparity can be attributed to both biological factors, such as hormonal differences, and psychosocial dynamics, including the variations in social support available to different sexes [23,24].

While our findings highlight that recurrent falls are significantly associated with an increased likelihood of depression, it is also possible that undocumented pre-existing depression contributes to a heightened likelihood of falling. Depression is known to reduce physical activity, impair concentration, and diminish vigilance during daily activities, all of which can increase fall risk [11]. This bidirectional relationship aligns with prior research indicating that individuals with depression are more prone to falls due to reduced motor coordination [11]. To address this complexity, our Kaplan-Meier analysis controlled for pre-existing depression by excluding individuals with a documented history of depression before the initial fall. This ensures that the findings primarily reflect depression as a consequence of falls rather than as a predisposing factor. However, the cyclical nature of falls and depression warrants further exploration to identify potential intervention points.

Our findings also shed light on the racial disparities that exist within this context. White participants report higher rates of recurrent falls, potentially linked to a greater prevalence of osteoporosis that increases susceptibility [25]. While the ‘Other Race’ category exhibits elevated depression rates, it is important to note that this group likely represents a heterogeneous population with varying experiences and challenges. Without detailed information on the specific racial and ethnic identities within this category, it is challenging to draw definitive conclusions about the underlying causes of these disparities. Further research is needed to understand the unique experiences of individuals classified within ‘Other Race’, including potential barriers to mental health care, social stressors, or other factors that may influence mental health outcomes following falls.

The psychological burden of recurrent falls is particularly profound. Adults aged 65–69 often confront the reality of aging, leading to the highest depression rates across fall groups. For example, the incidence of depression increases from 0.1163 following initial falls to 0.2143 after recurrent falls, clearly illustrating the escalating psychological toll. Furthermore, females exhibit a prevalence of depression reaching 0.5345 in the recurrent fall cohort, while those classified as ‘Other Race’ face disproportionately high depression rates, particularly after multiple falls.

These observations reinforce the notion that younger elderly adults are especially sensitive to the psychological ramifications of physical trauma [26]. The higher prevalence of depression among females reflects patterns noted in other studies, where women may be more affected by health declines due to a combination of hormonal shifts and social factors [23]. The elevated rates of depression within racial minority groups further highlight the added mental health burdens that these populations face, influenced by systemic healthcare barriers and the ongoing stressors encountered during recovery.

As we delve deeper, the link between repeated falls and depression risk becomes increasingly apparent. Older adults with recurrent falls are significantly more likely to experience depression than those who have fallen only once. Research indicates that multiple falls contribute to feelings of vulnerability and social withdrawal, which can worsen depressive symptoms [27]. Our findings suggest that individuals with recurrent falls face nearly 50% higher odds of developing depression, underscoring the cumulative impact of falls on both physical stability and mental health. This reality reinforces the urgent need for interventions that address the dual challenges of fall prevention and emotional support for older adults.

Adding another layer of complexity is the significant role that anxiety disorders play in this equation. The presence of anxiety dramatically increases the risk of developing depression following a fall, with a hazard ratio of 3.036 indicating that older adults with anxiety are over three times more likely to experience depression after falling. This relationship highlights the intricate interplay between psychological issues and the physical risks associated with falls [28]. Individuals grappling with anxiety may experience heightened fear surrounding mobility, creating a vicious cycle where the fear of falling exacerbates depressive symptoms.

Incorporating these insights into comprehensive intervention strategies is essential. By addressing both psychological challenges and fall risks, we can significantly improve outcomes for older adults. Preventing falls not only reduces the risk of physical injuries but also mitigates the likelihood of depression and other medical comorbidities, such as postoperative cognitive decline, delirium, and dementia, which are prevalent in this age group. Recognizing the dual impact of anxiety and recurrent falls allows healthcare providers to tailor support and resources that focus on preventing falls while also addressing the psychological burden.

This integrated approach should include evidence-based strategies such as exercise programs that improve balance, strength, and mobility, which have been shown to reduce fall risk. Routine mental health screenings following a fall are equally important to identify early signs of depression or anxiety, enabling timely interventions. Multidisciplinary care teams—including physiotherapists, geriatricians, and psychologists—can collaborate to address both the physical and psychological consequences of falls, providing a holistic framework for recovery. Additionally, community-based initiatives, such as support groups or educational workshops, can help build resilience, foster social connections, and reduce anxiety about falling. Home safety modifications, cognitive therapy, and ongoing health education further enhance these efforts, ensuring a supportive environment that promotes both physical and mental well-being.

By applying these tailored interventions, healthcare providers can better address the complex and interconnected challenges faced by older adults, ultimately improving recovery outcomes and enhancing their overall quality of life.

### Strengths

Our study leverages a robust dataset from the TriNetX network, allowing for detailed demographic and clinical analyses of older adults who experienced falls, which is a significant strength as it ensures the reliability and comprehensiveness of our findings. Additionally, the use of advanced statistical methods, including the Cox Proportional Hazards Model and Kaplan-Meier analysis, provides nuanced insights into the factors influencing depression post-fall, particularly distinguishing between initial and recurrent falls, thereby contributing valuable information to geriatric mental health research.

### Limitations

This study’s retrospective design limits the ability to fully establish causality between falls and subsequent depression. While we accounted for pre-existing depression as a general condition to isolate its impact on fall outcomes, we did not control for specific subtypes of depression, which may exert varying influences on fall risk due to differences in symptomatology, such as psychomotor retardation or cognitive impairment. Furthermore, the generalizability of our findings may be constrained by the use of data from a single healthcare system, Virginia Commonwealth University Health System (VCUHS). Regional or healthcare-system-specific differences, such as variations in patient demographics, healthcare delivery, and access to mental health resources, could influence the observed associations.

Additionally, potential biases associated with electronic health records, including incomplete documentation and unmeasured confounders such as socioeconomic status, medication use, and personal resilience factors, could affect the accuracy and applicability of the results. Future research should aim to replicate these analyses in diverse populations and healthcare systems to validate and extend our findings, ensuring their relevance to broader and more varied contexts. Prospective study designs could further enhance understanding by exploring the temporal relationships and underlying mechanisms linking falls and depression in older adults.

### Future Research Directions

Our future research will prioritize longitudinal studies to track mental health outcomes over time, with a specific focus on the trajectory of depression between the first and second falls. These studies will aim to provide a deeper understanding of how depression develops and evolves following recurrent falls, offering valuable insights into potential intervention points. We also plan to investigate the influence of factors such as specific subtypes of depression, socioeconomic status, and access to mental health resources. By addressing these areas, we aim to refine strategies that mitigate both the physical and psychological impacts of falls, ultimately informing targeted, evidence-based interventions to improve outcomes for older adults.

## CONCLUSION

This study underscores the significant association between recurrent falls and depression among older adults, revealing the interconnected impacts of physical and psychological health. By addressing demographic, social, and medical factors, tailored interventions can mitigate these challenges and improve outcomes. Future research should focus on longitudinal studies to deepen our understanding of these relationships, ultimately informing more effective prevention and recovery strategies for this vulnerable population.

## Figures and Tables

**Table 1.** ICD-10 codes for falls and recurrent fall in older adults.

Condition	ICD-10 Code	Description
Fall on same level from slipping, tripping and stumbling	W01.0	Accidental fall due to slipping, tripping, or stumbling
Fall from stairs and steps	W10.9	Unspecified fall from stairs or steps
Fall on and from ladder	W11	Accidental fall from ladder
Fall on and from scaffolding	W12	Accidental fall from scaffolding
Fall on and from building or structure	W13	Accidental fall from a building or structure
Other fall on same level	W18.30	Other falls on the same level
Other fall from one level to another	W17.89	Other falls from one level to another
Fall, unspecified	W19	Unspecified fall
Other specified fall	W18.49	Other specified falls
Fall from furniture	W08	Accidental fall from furniture, such as a bed or chair
Recurrent falls	R29.6	Recurrent falls occurring frequently over time
Anxiety Disorder	F41.9	Generalized anxiety disorder, unspecified, capturing broad symptoms of anxiety without specific focus
Depression	F32.x	Encompasses all subcategories of F32, indicating episodes of depression ranging from mild to severe

**Table 2.** Demographic distribution of older adult patients experiencing initial and recurrent falls.

Variables	Categories	Cohort 1 (Fall) *n* = 2710	Cohort 2 (Recurrent Fall) *n* = 1050	Bassline Comparison	
				*P*-Value	Standard Difference
Age	Current Age (Mean ± SD)	76.1 ± 6.87	76.0 ± 6.83	0.7023	0.0139
	Age at Index (Mean ± SD)	74.8 ± 6.88	74.6 ± 6.85	0.5622	0.0211
Sex	Female	1580	590	0.2395	0.0427
	Male	1140	470	0.1340	0.0544
	Unknown				
Race	White	1740	710	0.0488	0.0720
	Black or African American	830	290	0.0704	0.0662
	Unknown Race	80	30	0.8769	0.0057
	Other Race	50	20	0.9032	0.0044
	Asian	20	10	0.5074	0.0234
	American Indian or Alaska Native	10	10	0.0274	0.0721
	Native Hawaiian or Other Pacific Islander	0	10	<0.0001	0.1387

**Table 3.** Incidence and prevalence rates for older adults with initial fall and depression.

Variable	Category	Incidence Proportion	Prevalence	Incidence Rate (cases/person-day)
Overall		8.955%	32.472%	0.000353
Age	65–69	0.1163	0.3559	0.000473
	70–74	0.0870	0.3333	0.000347
	75–79	0.0682	0.3167	0.000269
	80–84	0.1111	0.2889	0.000430
	85 and older	0.0952	0.2692	0.000393
Sex	Female	0.0917	0.3734	0.000351
	Male	0.0968	0.2632	0.000399
Race	White	0.1016	0.3391	0.000413
	Black or African American	0.0656	0.3133	0.000241
	Asian	0.0	0.5	0.0
	American Indian/Alaska Native	0.0	0.0	0.0
	Native Hawaiian/Pacific Islander	0.0	0.0	0.0
	Other Race	0.25	0.4	0.001149
	Unknown Race	0.1429	0.25	0.000626

**Table 4.** Incidence and prevalence rates for older adults with recurrent falls and depression.

Variable	Category	Incidence Proportion	Prevalence	Incidence Rate (cases/person-day)
Overall		16.418	45.192	0.000687
Age	65–69	0.2143	0.5455	0.000902
	70–74	0.1765	0.4583	0.000775
	75–79	0.1429	0.5000	0.000605
	80–84	0.1667	0.4118	0.000746
	85 and older	0.1250	0.4000	0.000623
Sex	Female	0.1818	0.5345	0.000783
	Male	0.1429	0.3696	0.000599
Race	White	0.1818	0.4857	0.000780
	Black or African American	0.1579	0.4483	0.000655
	Asian	1.0	1.0	0.006831
	American Indian/Alaska Native	0.0	0.0	0.0
	Native Hawaiian/Pacific Islander	0.0	0.0	0.0
	Other Race	0.5	0.5	0.002728
	Unknown Race	0.3333	0.3333	0.001595

**Table 5.** Cox proportional hazards modeling analysis.

Variable	Hazard Ratio (HR)	Coefficient	Standard Error (SE)	Z Score	*P*-value	95% Confidence Interval (CI)
Outcome: Depression						
Recurrent vs. Initial Fall	1.488	0.397	0.078	5.087	<0.0001	(1.277, 1.734)
Sex (Male) vs. Female	0.866	−0.143	0.079	−1.820	0.0687	(0.742, 1.011)
Age at Index (Per Year Increase)	0.987	−0.013	0.006	−2.381	0.0172	(0.976, 0.998)
Anxiety Disorder	3.036	1.11	0.083	13.444	<0.0001	(2.582, 3.569)

## Data Availability

The data that support the findings of this study are available upon reasonable request. Interested researchers can obtain access to the data by submitting a formal request to the corresponding author at Asmaa.namoos@vcuhealth.org. The data is not publicly available due to privacy or ethical restrictions.
